# Human antibody recognition of H7N9 influenza virus HA following natural infection

**DOI:** 10.1172/jci.insight.152403

**Published:** 2021-10-08

**Authors:** Iuliia M. Gilchuk, Sandhya Bangaru, Nurgun Kose, Robin G. Bombardi, Andrew Trivette, Sheng Li, Hannah L. Turner, Robert H. Carnahan, Andrew B. Ward, James E. Crowe

**Affiliations:** 1 Vanderbilt Vaccine Center and; 2 Department of Pathology, Microbiology and Immunology, Vanderbilt University Medical Center, Nashville, Tennessee, USA.; 3 Department of Medicine, School of Medicine, University of California, San Diego, La Jolla, California, USA.; 4 Department of Integrative Structural and Computational Biology, Scripps Research Institute, La Jolla, California, USA.; 5 Department of Pediatrics, Vanderbilt University Medical Center, Nashville, Tennessee, USA.

**Keywords:** Infectious disease, Immunoglobulins, Influenza

## Abstract

Avian H7N9 influenza viruses cause sporadic outbreaks of human infections and threaten to cause a major pandemic. The breadth of B cell responses to natural infection and the dominant antigenic sites recognized during first exposure to H7 HA following infection are incompletely understood. Here, we studied the B cell response to H7 HA of 2 individuals who had recovered from natural H7N9 virus infection. We used competition binding, hydrogen-deuterium mass spectrometry, and single-particle negative stain electron microscopy to identify the patterns of molecular recognition of the antibody responses to H7 HA. We found that circulating H7-reactive B cells recognized a diverse antigenic landscape on the HA molecule, including HA head domain epitopes in antigenic sites A and B and in the trimer interface-II region and epitopes in the stem region. Most H7 antibodies exhibited little heterosubtypic breadth, but many recognized a wide diversity of unrelated H7 strains. We tested the antibodies for functional activity and identified clones with diverse patterns of inhibition, including neutralizing, hemagglutination- or egress-inhibiting, or HA trimer–disrupting activities. Thus, the human B cell response to primary H7 natural infection is diverse, highly functional, and broad for recognition of diverse H7 strains.

## Introduction

Influenza A viruses (IAVs) are categorized based on their surface proteins, HA and neuraminidase (NA), into 16 and 9 subtypes, respectively (1). H7 IAVs share the same HA subtype, which has been detected in wild birds in combination with N1 to N9 (2). H7Nx viruses can infect a broad range of species, from wild bird species to poultry and mammals, including seals, pigs, horses, and humans (3, 4).

Subtype H7 avian origin IAVs paired with N2, N3, N4, N7, or N9 NAs have caused more than 1567 confirmed human infections since 2003 (5, 6). The single H7N9 subtype had caused at least 616 deaths in humans since it emerged in 2013 (7, 8). Previous serology studies detected relatively robust antibody responses against the H7 HA after H7N9 infection that decreased but remained detectable after several years (9–12). Phylogenetic analyses of H7Nx viruses based on the H7 HA gene deduced 2 major genetic lineages, designated North American and Eurasian (13). However, results from antigenic cartography studies (14), results from H7Nx vaccine trials (15, 16), and the available data for the features of murine monoclonal antibodies (mAbs) elicited by H7 vaccination (17–19) suggest that H7Nx viruses are less antigenically diverse than seasonal influenza virus strains. Moreover, protective nearly pan-H7 subtype mAbs have been isolated from humans following natural H7N9 infection, for example mAbs HNIgGA6 (20), P52E03 (21), or H7-200 (22). These mAbs recognize the receptor binding site (RBS), HA antigenic site A, or trimer interface site II (TI-II) epitopes on the H7 head domain. Although antigenic diversity has been reported for Eurasian H7N9 viruses (11, 23), H7N9 infection also induces heterosubtypic responses in the polyclonal antibody repertoire against influenza type A group 1 and group 2 HAs (24). Heterosubtypic protective responses to H7N9 are driven partly by preexisting immunity against seasonal influenza H3N2 viruses, which are related at the genotypic and antigenic level to the H7 HA. This response includes HA stem-targeting human mAbs (25). Accordingly, H7N9 disease in humans is associated with decreased mortality in those born after 1968, which correlates with the time that seasonal H3N2 viruses first appeared in the human population (26). These snapshots of human B cell immunity to H7N9 infection are intriguing; however, comprehensive knowledge regarding the breadth, targeted epitopes, and functional activities of H7-reactive mAbs elicited by natural H7N9 infection remains limited.

## Results

**Figure 1:**
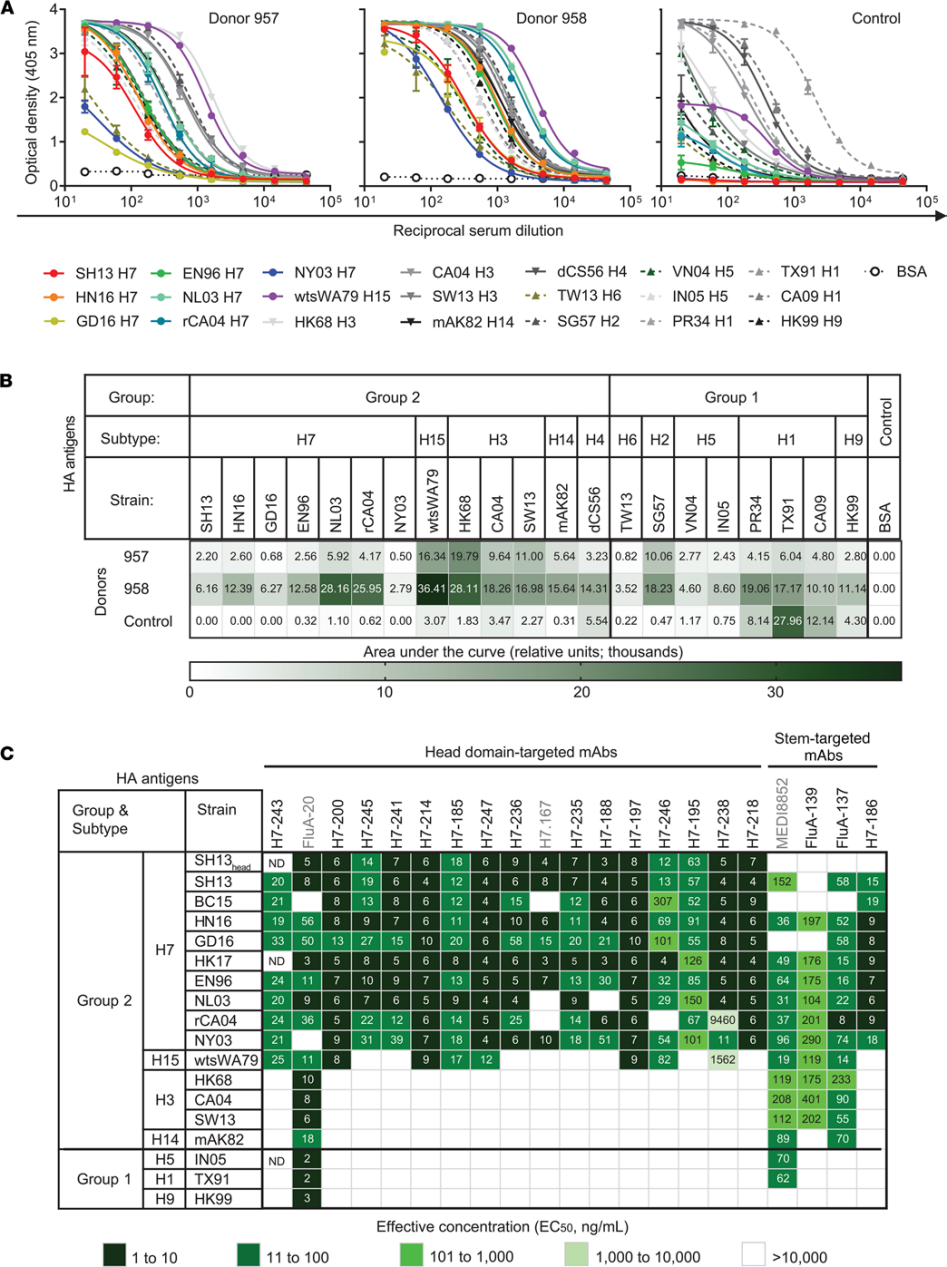
Natural H7N9 influenza virus infection elicits HA-specific antibodies with broad reactivity within the H7 subtype, mainly targeting the head domain. Cross-reactivity of antibodies to recombinant HA proteins from group 1 and 2 virus HA antigens as measured by ELISA. HAs were clustered by amino acid relatedness in the sequence phylogeny. (**A**) Dose-dependent HA antigen binding curves of serum samples from donors with naturally occurring laboratory-confirmed H7N9 infection (donors 957 and 958) or serum from control (an individual without exposure history to H7N9). Data are shown as mean ± SD of assay triplicates. (**B**) Heatmaps for cross-reactivity of polyclonal antibodies in convalescent serum samples from 2 H7N9 infection survivors or in negative control serum. Data represent the area under the curve for the binding assay. (**C**) Heatmaps for cross-reactivity of mAbs from the 2 survivors, isolated based on reactivity with the recombinant SH13 H7 HA antigen. Representative EC_50_ values (ng/mL) from 2 independent experiments are plotted. Three control mAbs are indicated by gray color. See also Supplemental Figure 1 and Supplemental Tables 1 and 2. ND, not determined.

We obtained peripheral blood mononuclear cells (PBMCs) and blood samples from 2 individuals born before 1968, approximately 11 months after recovery from naturally occurring laboratory-confirmed H7N9 influenza virus infection acquired in China (27). To determine the reactivity of anti-HA human antibodies, we first assessed binding of serum samples from H7N9 survivors to diverse recombinant HA proteins from group 2 (H7, H15, H3, H14, and H4) or group 1 (H6, H2, H5, H1, and H9) human and avian IAV. Serum from each of the survivors showed high reactivity to both group 1 and group 2 HA antigens, in contrast to control human serum from an individual without H7 virus exposure, which mainly reacted with human H1 IAVs from group 1 (Figure 1, A and B). This finding agrees with a previous report (24).

PBMCs were transformed with Epstein-Barr virus and other B cell stimuli, as previously described (28, 29), and the average number of cell clusters forming transformed B lymphoblastoid cell lines (LCLs) per well was determined. ELISA was used to screen cell supernatants for binding of human antibodies in LCL supernatants to H7 HA antigen from the H7N9 A/Shanghai/02/2013 (SH13) virus strain. H7-reactive LCLs were used for human hybridoma cell line development, as previously described (28). A panel of 18 cloned hybridoma cell lines secreting anti-H7 HA human mAbs was isolated, including 7 lines from the first individual (donor 957) and 11 from the second (donor 958). All mAbs were independent clones that displayed a high degree of antibody variable gene sequence diversity, including a unique heavy chain complementarity-determining region 3 sequence for each mAb, varying in length from 10 to 22 amino acids (Supplemental Table 1; supplemental material available online with this article; https://doi.org/10.1172/jci.insight.152403DS1). All mAbs exhibited low mutation frequencies in their antibody variable gene sequences, consistent with the retention of clones in the memory B cell compartment that are typical of a primary immune response following first infection.

The isolated anti-H7 mAbs bound in ELISA to the corresponding recombinant H7 HA protein of the infecting isolate (previously reported as A/British Columbia/1/2015 H7N9, BC15) and to the closely related SH13 strain HA used in LCL screening (Figure 1C). The majority (14 of 17) of mAbs also recognized a recombinant protein representing the head domain of SH13 HA (Figure 1C, Supplemental Figure 1, and Supplemental Table 2). In addition, the mAbs bound to recombinant HA molecules from representative H7 strains that are more distantly related, including: 1) Eurasian H7 viruses: A/England/268/1996 H7N7 (EN96), A/Netherlands/219/2003 H7N7 (NL03), Yangtze River Delta sublineage A/Hunan/02650/2016 H7N9 (HN16) and A/Hong Kong/125/2017 (HK17), and Pearl River Delta sublineage A/Guangdong/17SF003/2016 H7N9 (GD16); and 2) North American H7 viruses: A/Canada/rv504/2004 H7N3 (rCA04) and A/New York/107/2003 H7N2 (NY03) (30–32). A large fraction (8 of 15) of head domain–reactive mAbs also recognized H15 HA from the A/wedge-tailed shearwater/Western Australia/2576/1979 H15N9 (wtsWA79). MAb FluA-137, the broadest antibody from the panel, cross-reacted with H3 HAs from A/Hong Kong/1/1968 H3N2 (HK68), A/California/07/2004 H3N2 (CA04), A/Switzerland/9715293/2013 H3N2 (SW13), and H14 HA from A/mallard duck/Astrakhan/263/1982 H14N5 (mAK82) (Figure 1B and Supplemental Figure 1). None of the mAbs from the panel cross-reacted with representative HA proteins from group 1 subtypes H1, H5, H9, etc. (Supplemental Table 2). In summary, the H7-reactive human mAbs elicited in each survivor by natural H7N9 infection mainly recognized the H7 head domain and exhibited broad reactivity within the H7 subtype. Heterosubtypic reactivity for the mAbs targeting the H7 head domain primarily involved recognition of H15 HA.

**Figure 2:**
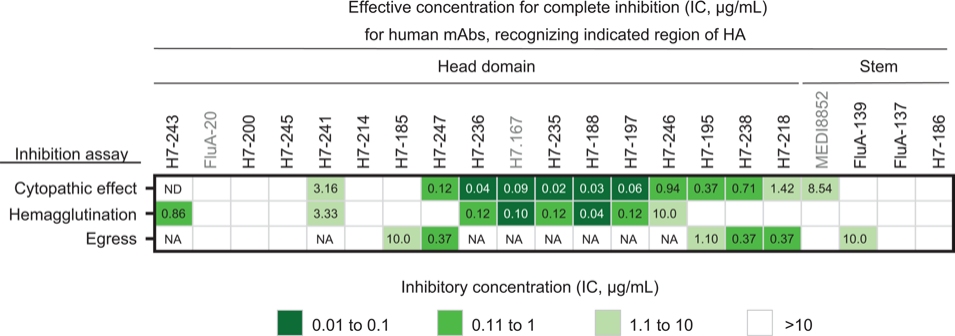
Anti-H7 human mAbs inhibit H7N9 virus by diverse mechanisms. Individual mAbs were assessed for H7N9 virus neutralization using assays for inhibition of cytopathic effect (CPE), hemagglutination inhibition (HAI), and egress inhibition. Representative EC_100_ values (μg/mL) from 2 independent experiments are plotted as a heatmap. Data represent 1 of 2 independent experiments. Three control mAbs are indicated by gray color. See also Supplemental Figures 2 and 3. NA, not applicable.

The influenza HA protein mediates multiple functions in the virus life cycle, including binding the virus to sialic acid on the surface of target cells (attachment) and fusing the virus envelope with the late endosomal membrane. Anti-HA antibodies can interfere with virus replication at different stages in the life cycle of the influenza virus, such as by inhibiting receptor binding and membrane fusion or functions associated with the viral NA like egress and release (33). To determine the potency of neutralizing activity of these H7-reactive mAbs, we developed and used a sensitive functional assay measuring antibody-mediated inhibition of virus-induced cytopathic effect (CPE). The real-time cell analysis (RTCA) assay quantifies virus-induced CPE caused by SH13 H7N9 IDCDC-RG32A virus in cell culture monolayers using cell impedance. This assay design monitors multicycle virus replication and detects neutralizing activity at any stage in the virus life cycle. The majority (10 of 17) of tested mAbs from the panel exhibited neutralizing activity in the RTCA assay, as measured by protection against CPE (Figure 2, Supplemental Figure 2A, and Supplemental Figure 3A).

To determine the specific stage in the virus life cycle and the mechanism of inhibitory action for the neutralizing anti-H7 mAbs, we next performed HAI assays with the SH13 H7N9 IDCDC-RG32A virus. More than half (8 of 18) of mAbs exhibited HAI activity (Figure 2, Supplemental Figure 2B, and Supplemental Figure 3B). Of note, the most potently neutralizing antibodies in the panel inhibited the virus at the attachment stage, according to the HAI data. Inhibition of egress of virions from infected cells in vitro was tested using a previously described assay (34, 35), which quantified newly produced viral particles released into the supernatant by an HA assay. Since mAb-mediated HAI activity interferes with this test, we excluded the 8 mAbs with HAI activity from this analysis. About half (6 of 11) of the tested mAbs inhibited influenza virus at the egress stage, as evidenced by eliminating H7N9 virus release into infected cell supernatants (Figure 2, Supplemental Figure 2C, and Supplemental Figure 3C). Egress-inhibiting antibodies identified in the panel are mainly head domain–targeting mAbs. Here, we describe an additional mechanism of viral neutralization for H7-specific mAbs.

**Figure 3:**
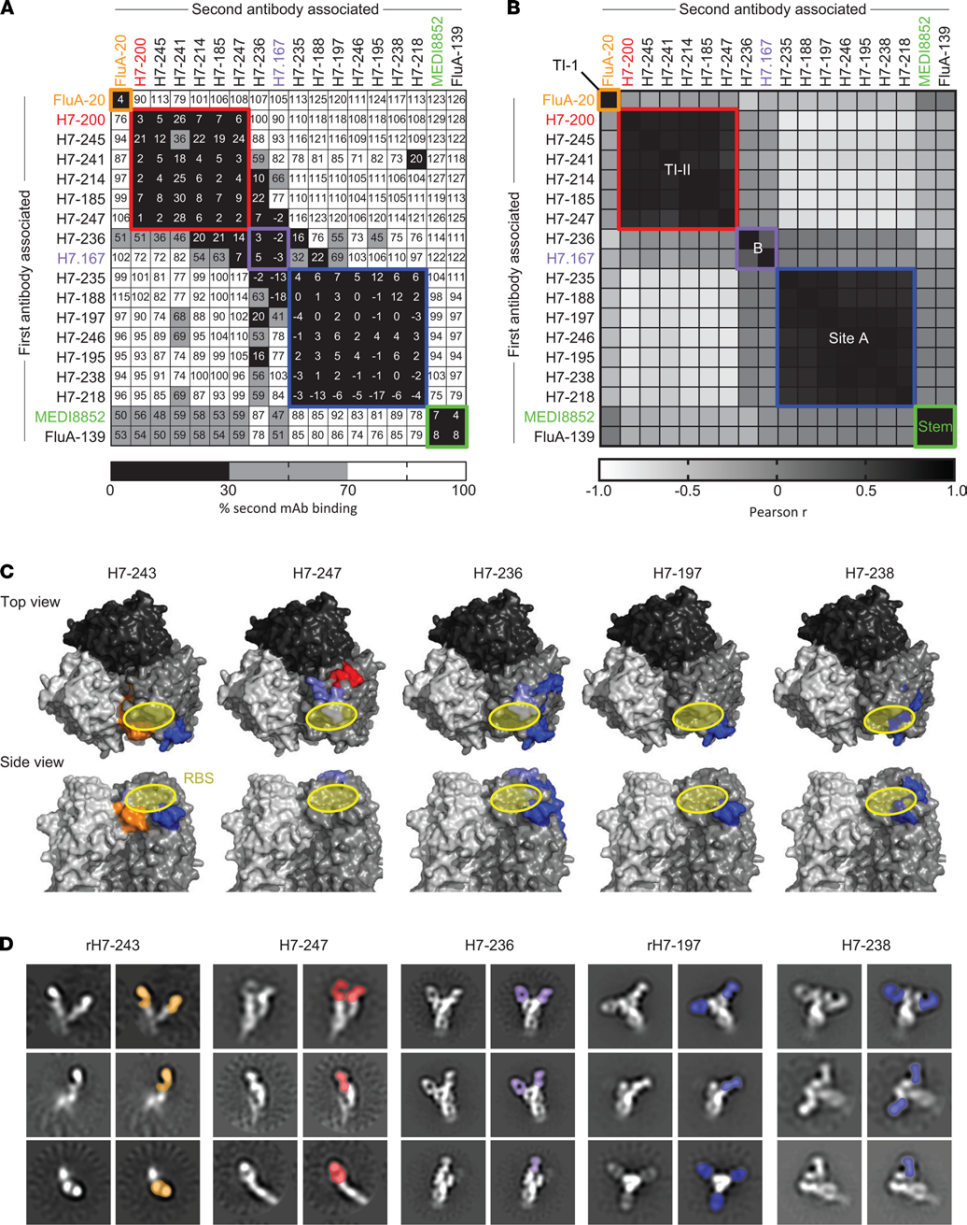
Natural H7N9 influenza virus infection elicits HA-specific mAbs targeting 3 main regions on H7 head (sites A and B, HA interface) and stem domains. MAbs were assessed for competition binding by biolayer interferometry (BLI) using a Bio-Rad device with trimeric HA from SH13 H7N9 virus strain. MAbs were judged to compete for the same site if the maximum binding of the second antibody was reduced to no more than 30% of its uncompeted binding (shown in black boxes). The mAbs were considered noncompeting if the maximum binding of the second mAb was at least 70% of its uncompeted binding (shown in white boxes). Gray color indicates an intermediate phenotype (competition between 30% and 70% of uncompeted binding). Orange, red, violet, blue, and green boxes indicate inferred competition-binding groups. Reference mAbs with known epitopes are indicated with colors. (**A**) BLI raw data; (**B**) Pearson’s correlation coefficient for BLI data; (**C**) HDX-MS profiles of H7-243, H7-247, H7-236, H7-197, or H7-238 mAbs were mapped onto the surface of the H7 HA trimer (Protein Data Bank ID 4N5J). The yellow circle indicates the RBS. Amino acid residues within the epitope with decreased deuteration level upon H7-243, H7-247, H7-236, H7-197, or H7-238 mAbs binding are indicated by colors corresponding to competition-binding groups: orange, red, violet, and blue. (**D**) Two-dimensional (2D) class averages of H7 HA in complex with anti-H7 Fabs. For each complex, 3 different views of the 2D class averages are shown on the left column with corresponding images with the Fabs colored in the right column. rH7-243 caused the HA trimer to fully decompose into HA protomers, while rH7-247 did so only partially under the given experimental conditions. See also Supplemental Figures 4 and 5.

We next defined groups of mAbs that bind to common major antigenic sites using a competition-binding assay with recombinant trimeric SH13 HA ectodomain (Figure 3, A and B). The assay was based on the detection of sequential protein binding and performed using an Octet RED96 BLI device (FortéBio). Epitopes for the influenza H7 HA-reactive mAbs H7.167 (36), FluA-20 (34), H7-200 (22), and MEDI8852 (37) were reported previously. We used these mAbs as known epitope controls to map the antigenic sites on the HA surface by determining competition-binding groups for the newly isolated H7 mAbs. The 15 mAbs from the panel fell into 4 competition-binding groups: 3 on the head domain (site A, site B, and the TI-II site) and 1 on the stem domain. Interestingly, most head domain–targeted mAbs from both survivors (13 of 14 tested) recognized 1 of 2 epitopes: site A or TI-II. Site A is a conserved and well-known target for H7-specific mAbs (21), whereas only a single mAb, designated H7-200 (22), has been reported previously to recognize the TI-II site. The H7-specific mAbs that possessed HAI activity segregated mainly into 2 competition-binding groups, corresponding to antigenic sites A and B. The exception is the HAI-active mAb H7-241 that recognized the head domain surface within the site TI-II competition-binding group. MAbs that possessed egress-inhibiting activity segregated among different competition-binding groups. The non-neutralizing head domain–targeted mAbs comprised a competition-binding group with the mAb H7-200 known to bind the TI-II site of the HA head domain. MAb FluA-139 competes with the HA stem–targeting mAb MEDI8852. Two other mAbs, FluA-137 and H7-186, likely also recognize the stem region since they did not react with the H7 head domain protein in ELISA. The putative stem epitopes for FluA-137 and H7-186 likely are distinguished from that for MEDI8852 because these mAbs did not recognize HA trimer protein on a biosensor surface. The pan-H7 and heterosubtypic mAbs reacting with H7 and H15 subtypes equally segregated among different competition-binding groups on the head domain. The heterosubtypic mAbs recognizing H7, H15, and H3 HAs are all stem-targeted mAbs, consistent with previous findings (23–25).

Next, we conducted studies to confirm the inferred location of the sites corresponding to competition-binding groups with 2 complementary structural analyses: hydrogen-deuterium exchange mass spectrometry (HDX-MS) and negative stain electron microscopy (nsEM) of H7/mAb complexes (Figure 3, C and D, Supplemental Figures 4 and 5). Results for representative mAbs from both HDX-MS proteomics studies and nsEM structural studies agreed with the assignment of competition-binding groups as determined by BLI. The HDX-MS data revealed putative epitopes for all 5 representative mAbs that contact different locations near the RBS. Studies with nsEM showed minor differences in the putative epitopes for the H7-197 and H7-238 mAbs, which both belong to the site A competition-binding group. MAb H7-197 binds near the RBS, while the epitope for mAb H7-238 is located on the side of the HA head domain below the RBS. This difference explains why H7-197 possesses HAI activity, but H7-238 does not. In addition, nsEM studies showed that the neutralizing mAbs H7-243 and H7-247 have an additional functional phenotype. These mAbs disrupt HA trimers when mixing occurs before nsEM imaging, as previously reported for the protective but non-neutralizing TI-I site–specific mAb FluA-20 (34) and the TI-II site–specific mAb H7-200 (22).

## Discussion

Serology studies have shown that human H7N9 virus infection stimulates the induction of H7-reactive antibody responses that persist for at least several years (10, 11, 24, 38). Kinetic monitoring of such responses revealed that heterosubtypic H7 and H3 binding/neutralizing antibody responses typically appear and peak earlier than H7 subtype responses, likely mediated by memory recall of H3-reactive clones (24). Furthermore, antibodies reacting with the H7 HA stem region were induced early after infection, whereas antibodies against the H7 head domain appeared later after infection. Finally, the mean HAI antibody levels in survivors peaked 3 months after infection, then declined to a titer of 80 at 11 months or titer of 40 at 22 months after infection. Neutralizing antibody titers increase slower than HAI antibody titers but continue to increase until 35 months after illness onset (11). Thus, the specificity and breadth of the human antibody response to H7 HA changes over time. MAb studies of the human response to H7N9 infection were conducted previously only using plasmablasts circulating in blood during the acute phase of infection (23). Here, we investigated the H7-reactive human mAb response against H7N9 infection that persisted months after infection, in studies based on the isolation of H7-reactive mAbs from human peripheral blood memory B cells obtained approximately 11 months after natural H7N9 infection. Overall, our results are closely related to those of some previous anti-H7 mAb repertoire studies for H7N9 survivors (23) or vaccinees (36, 39), which suggests that natural H7N9 infection and vaccination induce similar repertoires of B cells. The data here suggest that memory B cell–derived mAbs show more substantial cross-reactivity against heterologous H7 HAs than antibodies isolated early after infection or after vaccination. Most head domain–targeted mAbs isolated 11 months after infection retained reactivity against all H7Nx viruses tested in a panel representing all H7 virus lineages described to date. A good proportion of the head-targeted mAbs also showed cross-reactivity to H15, a group 2 HA closely related to H7 HAs. In contrast to mAbs derived from survivor plasmablasts, where H7/H3 cross-reactive mAbs represent one of the major antibody classes, we found only 2 mAbs bound to both H7 and H3 HAs. Of note, the panel of H7-reactive mAbs was isolated from PBMC samples of survivors born before 1968, whereas the previous H7/H3 cross-reactive plasmablast-derived mAbs were isolated only from survivors born after 1968. The same group (23) did not reveal any cross-reactive H7/H3 mAb from a survivor born before 1968. A limitation of our studies is that they were performed with B cells from only 2 representative donors, and we obtained a relatively small panel of isolated mAbs. However, the breadth of reactivity of serum antibodies in these 2 survivors is consistent with that shown for other H7N9 survivors in a previous serologic study (24).

Previously, structure-function analysis of antibody repertoires induced by natural H7N9 infection was mostly limited to characterization of potently neutralizing mAbs (20, 21, 23). We found, like others, the most potent anti-H7 neutralizing antibodies possess HAI activity and target the RBS and/or antigenic site A adjacent to the HA RBS. We also demonstrated that anti-H7 HA neutralizing mAbs often exhibit some level of egress inhibition activity. The most potent egress inhibitors from the panel (H7-247, H7-238, and H7-218) completely inhibited egress at concentration 370 ng/mL. In addition to anti-NA mAbs, this mechanism of protection was previously noted with an anti-H3 head mAb H3v-47 (35) and anti-H3 or -H1 stem mAbs (40). In comparison with the mAbs characterized here, the most potent previously reported anti-N9 NA mAbs exhibited IC_100_ values at approximately 5 ng/mL concentration (41), a head domain–targeting anti-H3 mAb (H3v-47) at 370 ng/mL, and anti-H3 or -H1 stem mAbs at greater than 64 μg/mL concentration range.

Several putative H7 antigenic sites (sites A, B, C, D, and E) on the HA globular head (42) and antigenic sites on the HA stem domain have been predicted for H7 HA because of the highly similar structure of the H7 stem to that of H3 subtype viruses. The HA antigenic sites are not discrete domains; rather, they are clusters of surface residues often recognized by antibodies. Nevertheless, H7-reactive mAbs reported to date recognize only the RBS and/or site A and B adjacent to the HA RBS, the TI-I site on the HA head, and the stem region. Here, we report that a large portion of the antibodies elicited by natural H7N9 infection that persist in the memory B cell compartment target the TI-II epitope or sites overlapping between the TI-II site and site B on the HA head. Many of the mAbs described here that appear to bind to TI regions exhibit an HA trimer disruption phenotype in nsEM studies.

Information on epitopes targeted by broadly reactive neutralizing and/or protective mAbs is a primary requirement for structure-based vaccine antigen design. Isolation and characterization of naturally elicited H7-specific antibodies allow us to confirm the importance of previously characterized accessible, surface-exposed, and vulnerable sites of H7 HA — site A, site B, and the stem region — and reveal an additional area of vulnerability, the TI-II site. On the other hand, this type of study can reveal that some sites of vulnerability for antibodies that are potent in neutralization and/or protection can only be recognized by a restricted set of antibodies with unusual features, like large numbers of somatic mutations in the variable gene sequences and complementarity-determining regions (CDRs) or extra-long or -short CDR3 loops. Such constraints may make development of protective immunity after vaccination more challenging in most people. The findings here are interesting in that antibodies identified after H7N9 infection in our work have low mutation frequencies in the variable gene sequences, and there is no requirement for especially short or long heavy chain CDR3 loops. Together with the high prevalence of virtually pan-H7Nx, broadly reactive antibodies in the panel isolated from H7N9 survivors’ memory B cells, these 2 considerations suggest a solid foundation for designing structure-based vaccines against avian H7Nx viruses. At the same time, the lack of reactivity to heterosubtypic HAs beyond H15 HA exhibited by the mAbs isolated suggests that the head domain site A, site B, and TI-II epitopes (or, at least the H7 ones) are not likely to be used successfully as the target for vaccine antigen design for universal influenza vaccine development.

In conclusion, these studies show that mAbs induced by naturally occurring H7N9 infection in humans exhibit broad cross-reactivity within the H7 and H15 subtypes, target mainly antigenic site A and the TI-II site on the H7 head domain, and neutralize virus by diverse mechanisms. Given that the antibody variable genes for the mAbs isolated here contained few somatic mutations, the results suggest these antibodies mostly comprised a primary immune response to this H7N9 infection. These findings help inform the development of universal or avian H7N9 influenza vaccines and offer tools to characterize and evaluate new antigens and candidate vaccine viruses.

## Methods

### Cell lines and viruses.

The reverse-genetics–derived virus with the H7N9 HA and NA genes on a PR8 backbone, designated A/Shanghai/2/2013 (H7N9)-PR8-IDCDC-RG32A (Influenza Reagent Resource, FR-1389), was propagated in embryonated chicken eggs (Charles River Laboratories) and manipulated under Biosafety Level 2 (BSL-2) conditions with BSL-3 practices. Virus titration and neutralization assays were performed on MDCK (ATCC, CCL-34) epithelial cells. Cells were determined to be mycoplasma free at laboratory passage 2 and monthly when in use.

### Recombinant protein expression and purification.

A cDNA encoding the HA genes from the IAVs A/Shanghai/02/2013, A/British Columbia/1/2015, A/Hunan/02650/2016, A/Guangdong/17SF003/2016, A/Hong Kong/125/2017 H7N9, A/England/268/1996 and A/Netherlands/219/2003 H7N7, A/Canada/rv504/2004 H7N3 and A/New York/107/2003 H7N2, A/shearwater/Western Australia/2576/1979 H15N9, A/Hong Kong/1/1968, A/California/07/2004, A/Switzerland/9715293/2013 H3N2, A/mallard duck/Astrakhan/263/1982 H14N5, A/duck/Czechoslovakia/1956 H4N6, A/Taiwan/2/2013 H6N1, A/Singapore/1/1957 H2N2, A/Vietnam/1203/2004 H5N1, A/Puerto Rico/8/1934, A/Texas/36/1991 and A/California/04/2009pnd H1N1, and A/Hong Kong/1073/1999 H9N2 were optimized for expression. The cDNAs were synthesized (GenScript) as soluble trimeric constructs by replacing the transmembrane and cytoplasmic domain sequences with cDNAs encoding the GCN4 trimerization domain and a His6-tag at the C-terminus. Synthesized genes were subcloned into the pcDNA3.1(+) mammalian expression vector (Thermo Fisher Scientific). HA protein was expressed by transient transfection of FreeStyle 293-F cells (Thermo Fisher Scientific, R79007) with polyethyleneimine transfection reagent and was grown in expression medium (FreeStyle 293 Expression Medium; Thermo Fisher Scientific). The supernatants were harvested after 7 days, filter-sterilized with a 0.4 μm filter, and purified with HisTrap TALON FF crude columns (GE Healthcare Life Sciences).

### MAb isolation.

Human hybridoma cell lines secreting anti-H7 mAbs were generated using methods as described previously (28, 29). Briefly, human B cells in the PBMC suspension were immortalized by transformation with Epstein-Barr virus in the presence of CpG10103, cyclosporin A, and a Chk2 inhibitor. On day 8, the supernatants from transformed B cells were used to screen for the presence of antigen-reactive antibodies against H7 HA from A/Shanghai/2/2013 (H7N9) using a capture ELISA described in detail below. Cells from the wells containing B cells secreting HA-reactive antibodies were fused with HMMA2.5 myeloma cells (provided by Marshall Posner, Dana-Farber Cancer Institute, Boston, Massachusetts, USA) using an ECM 2001 electro cell manipulator (BTX), and human hybridomas were selected in medium with hypoxanthine-aminopterin-thymidine solution containing ouabain. The hybridomas were subcloned by flow cytometric sorting of single cells into 384-well plates and then expanded in culture. The selected cell line was grown in hybridoma growth medium (ClonaCell-HY medium E from STEMCELL Technologies) and then switched to serum-free medium (Gibco Hybridoma-SFM, Thermo Fisher Scientific) for antibody expression. IgGs from the hybridoma cell line supernatants were purified by affinity chromatography using MabSelect SuRe columns (GE Healthcare Life Sciences, now Cytiva). Purified IgGs generated from hybridomas were used for all in vitro studies.

### Generation of antibody Fabs.

For the expression of recombinant rH7-243 and rH7-197 Fabs, the nucleotide sequences of antibody heavy and light chain antibody variable genes were codon optimized for mammalian expression and synthesized at Twist Biosciences. The resulting gene fragments were cloned directly at Twist Biosciences into the pTwist CMV BetaGlobin WPRE NEO mammalian expression vector (Twist Biosciences). MAb heavy/light chain constructs were used to transfect Expi293F cells (Thermo Fisher Scientific, A14528) transiently, and supernatants were harvested after culturing for 6 to 7 days. Recombinant Fabs were purified with Anti-CH1 CaptureSelect column (Cytiva). IgGs produced by the corresponding human mAb-secreting hybridoma cell lines in serum-free medium (Gibco Hybridoma-SFM, Thermo Fisher Scientific) were purified by affinity chromatography using HiTrap MabSelect SuRe column (GE Healthcare Life Sciences).

### IgG digestion.

MAb IgGs were incubated 3 to 4 hours with 4% w/w papain at 37°C. Then 0.5 M iodoacetamide was added to stop digestion. CaptureSelect IgG-Fc multispecies resin (Thermo Fisher Scientific) was added to the digested material and incubated overnight at 4°C. The supernatant was separated from Fc-bound resin with a 0.2 μm filter and then purified over a Superdex 200 size exclusion column (Cytiva). The Fab peak was concentrated for complexing with antigen.

### ELISA.

For screening ELISA, plates were incubated with culture supernatants from transformed B cells. For cross-reactivity assays, serum samples were assessed at 3-fold dilutions starting from 1/20, in triplicate, and purified mAbs were assessed at concentrations ranging from 10 μg/mL to 0.1 ng/mL, in triplicate. We performed ELISAs using 384-well plates coated overnight at 1 μg/mL with the recombinant HA protein of interest. The plates then were blocked with 50 μL of 5% nonfat dry milk and 0.1% Tween-20 in D-PBS (ELISA buffer) for 1 hour at room temperature. The plates were washed and serial 3-fold dilutions of the serum samples or mAbs were added to the wells and incubated for an hour. The plates were washed and 25 μL of ELISA buffer containing a 1:4000 dilution of anti-human IgG alkaline phosphatase conjugate (Meridian Life Science, W99008A) was added. After a final wash, 25 μL of phosphatase substrate solution (1 mg/mL p-nitrophenol phosphate in 1 M Tris aminomethane) was added to the plates, then incubated for 1 hour, and the optical density values were measured at 405 nm wavelength on a BioTek plate reader. The plates were washed 3 times between each step with PBS containing 0.05% Tween-20. The area under the curve was calculated in Prism software (GraphPad Software). EC_50_ values for mAbs were determined using Prism 5.0 software (GraphPad Software) after log transformation of antibody concentration using nonlinear regression analysis. Each experiment was conducted twice independently.

### HAI assay.

The HAI assay was performed with A/Shanghai/2/2013 (H7N9)-PR8-IDCDC-RG32A. For HAI, 25 μL of 4 HA units of virus were incubated for 1 hour at room temperature with 25 μL 2-fold serial dilutions of antibodies starting at 10 μg/mL in PBS. The 50 μL of antibody-virus mixture was incubated for 45 minutes at 4°C with 50 μL of turkey red blood cells (Rockland Immunochemicals) diluted in PBS. The IC_100_ value was defined as the lowest antibody concentration that inhibited hemagglutination of red blood cells.

### Egress inhibition assay.

MDCK cells were seeded in plain Dulbecco’s modified Eagle medium (Gibco DMEM, Thermo Fisher Scientific) containing 10% FBS in 96-well plates overnight. The cells were washed 3 times with Virus Growth Medium (VGM) (DMEM with 2% BSA and 2 μg/mL TPCK-treated trypsin, MilliporeSigma) and 100 μL of 1 multiplicity of infection (MOI) of A/Shanghai/2/2013 (H7N9)-PR8-IDCDC-RG32A virus in VGM added to the cells and incubated for 3 hours at 37°C in 5% CO_2_. The cells were then washed with VGM again and replenished with VGM containing 3-fold serial dilutions of mAbs or zanamivir (GlaxoSmithKline), starting at the highest concentration of mAbs 10 μg/mL or equimolar. The plates were incubated for 21 hours at 37°C in 5% CO_2_, and the supernatants were collected for performing the HA assay. For HA assay, we used turkey red blood cells (Rockland Immunochemicals) that were washed and diluted to 0.5% in PBS. A volume of 50 μL of the supernatants was incubated with 50 μL of the 0.5% turkey red blood cells in V-bottom plates for 1 hour at 4°C. The IC_100_ values were defined as the lowest antibody concentration added to infected MDCK cells that corresponded to absence of virus in supernatant according to HA of red blood cells.

### xCELLigence RTCA assay.

To screen neutralizing activity of mAbs, we used a high-throughput and quantitative RTCA and xCELLigence Analyzer (ACEA Biosciences Inc.) that assesses kinetic changes in cell physiology, including virus-induced CPE. Fifty microliters of cell culture medium (Opti-MEM supplemented with 2 μg/mL of TPCK-trypsin [MilliporeSigma, T1426] and penicillin/streptomycin [Gibco, Thermo Fisher Scientific, 10378-016]) was added to each well of a 96-well E-plate to obtain a background reading. Forty thousand (40,000) MDCK cells in 50 μL of cell culture medium were seeded per well, and the plate was placed on the analyzer. Measurements were taken automatically every 15 minutes, and the sensograms were visualized using RTCA software version 2.1.0 (ACEA Biosciences Inc). A/Shanghai/2/2013 (H7N9)-PR8-IDCDC-RG32A virus (0.25 MOI) was mixed with six 3-fold dilutions of mAbs starting at 10 mg/mL in a total volume of 100 μL and incubated for 1 hour at room temperature. At 22 to 24 hours after seeding the cells, the virus/mAb mixtures were added in 3 replicates to the cells in 96-well E-plates. Wells containing virus only in the absence of mAb and wells containing only MDCK cells in medium were included on each plate as controls. Plates were measured continuously (every 15 minutes) for over 48 hours to assess virus neutralization. Dose-dependent neutralization curves were graphed, and neutralization IC_50_ values were calculated using GraphPad Prism.

### NsEM.

Recombinant rH7-243 and rH7-197 and cleaved from hybridoma-derived IgG H7-247, H7-236, and H7-238 Fabs were incubated with uncleaved H7 SH13 HA or H7 Hunan trimer overnight at 4 times molar excess of Fab. Then rH7-243 and rH7-247 were incubated with HA for 20 seconds to catch an intact complex before degradation. The complex was added to carbon-coated 400 mesh copper grids and stained with 2% uranyl formate. Micrographs were collected on a 120 kV Tecnai Spirit microscope with a 4kx4k TemCam F416 camera using Leginon. Images were processed within Appion (43). Particles were selected with DoGpicker (44), and 2D classes were generated with multivariate statistical analysis/multireference alignment (45). Particles were false colored in Adobe Photoshop.

### Competition-binding groups.

BLI on an Octet Red instrument (FortéBio) was used to perform competition-binding assays. We loaded the H7 HA from A/Shanghai/2/2013 H7N9 with 6xHis-tag onto Anti-Penta-HIS biosensors (FortéBio, 18-5120) at a concentration of 10 μg/mL, then tested binding of 2 successively applied mAbs at 50 μg/mL concentration. Antigen and mAb dilutions were made in 1X Kinetics Buffer (FortéBio, 18-5032). The mAbs were defined as competing if the first Ab reduced binding of the second Ab by more than 70%. The mAbs were defined as noncompeting if the first Ab reduced binding of the second by less than 30%.

### Peptide fragmentation and HDX-MS.

To maximize peptide probe coverage, the optimized quench condition was determined prior to deuteration studies. The soluble trimeric H7 HA ectodomain was diluted with buffer of 8.3 mM Tris, 150 mM NaCl, in H_2_O (pH 7.5), at 0°C, and then quenched with 0.8% formic acid (v/v) containing various concentrations of GuHCl (0.8 to 6.4 M) and Tris(2-carboxyethyl)phosphine (TCEP) (0.1 or 1.0 M). After incubating on ice for 5 minutes, the quenched samples were diluted 3-fold with 0.8% formic acid (v/v) containing 16.6% (v/v) glycerol and then were frozen at –80°C until they were transferred to the cryogenic autosampler. Using the quench buffer of 6.4 M GuHCl, 1 M TCEP in 0.8% formic acid, gave an optimal peptide coverage map. The samples later were thawed automatically on ice and then immediately passed over an AL-20-pepsin column (16 μL bed volume, 30 mg/mL porcine pepsin; MilliporeSigma). The resulting peptides were collected on a C18 trap and separated on a C18 reversed-phase column (0.2 × 50 mm, Optimize Technologies Inc) running a linear gradient of 0.046% (v/v) trifluoroacetic acid, 6.4% (v/v) acetonitrile to 0.03% (v/v) trifluoroacetic acid, 38.4% (v/v) acetonitrile over 30 minutes with column effluent directed into an Orbitrap Elite mass spectrometer (Thermo Fisher Scientific). Data were acquired in both data-dependent MS:MS mode and MS1 profile mode. Proteome Discoverer software (Thermo Fisher Scientific) was used to identify the sequence of the peptide ions. HDEXaminer software (Sierra Analytics Inc.) was used for the analysis of the mass spectra. IgG-bound HAs were prepared by mixing IgGs with trimeric H7 HA at a 1:1.1 (HA/IgG) stoichiometric ratio. The mixtures were incubated at 25°C for 30 minutes. All functionally deuterated samples, except for the equilibrium-deuterated control, and buffers were prechilled on ice and prepared in the cold room. Functional deuterium-hydrogen exchange reaction was initiated by diluting free HA or antibody-bound HA stock solution with D_2_O buffer (8.3 mM Tris, 150 mM NaCl, in D_2_O, pDREAD 7. 5) at a 1:2 v/v ratio. At 10, 100, and 1000 seconds, the quench solution was added to the respective samples, and incubated on ice for 5 minutes, then diluted 3-fold with 0.8% formic acid before freezing at –80°C. In addition, nondeuterated samples, equilibrium-deuterated back-exchange control samples, were prepared as previously described (46, 47). The centroids of the isotopic envelopes of nondeuterated, functionally deuterated, and fully deuterated peptides were measured using HDEXaminer, then converted to corresponding deuteration levels with corrections for back exchange (48).

### Statistics.

Statistical calculations were performed with Prism software (GraphPad). For binding curves, data are shown as mean ± SD of assay triplicates. EC_50_ values for mAbs were determined using Prism software (GraphPad) after log transformation of antibody concentration using nonlinear regression analysis. Each experiment was conducted twice independently. Ab IC_50_ values were calculated using a formula based on the Spearman-Kärber equation (49). The Pearson’s correlation of each biotinylated mAb to each other biotinylated mAb was calculated using the median inhibition percentage from 3 different experiments using the *corr* method of the *Pandas* Python package (50). Hierarchical clustering was then performed on these Pearson’s correlations using the *clustermap* method of the *Seaborn* Python package. The clustering information was exported in newick format and imported into Interactive Tree of Life v4 (51), which was used to display a hierarchically clustered heatmap before importation into Excel (Microsoft) for final collection of values.

### Study approval.

A blood sample from 2 survivors of natural H7N9 infection, described in a case report previously (27), was collected from each individual after receipt of written informed consent approximately 11 months after recovery from infection. The study was approved by the Institutional Review Board of Vanderbilt University Medical Center.

## Author contributions

IMG, SB, ABW, RHC, and JEC planned experiments; IMG, SL, SB, NK, and HLT performed experiments; IMG and JEC wrote the manuscript; IMG, SB, NK, RGB, AT, SL, HLT, RHC, ABW, JEC reviewed, edited, and approved the final manuscript; and JEC and ABW obtained funding.

## Supplementary Material

Supplemental data

## Version 1 08/26/2021

In-Press Preview

## Version 2 10/08/2021

Electronic publication
