# Supplementary material for: Human antibody recognition of H7N9 influenza virus HA following natural infection
Source: JCI Insight. 2021 Oct 8;6(19):e152403. doi: 10.1172/jci.insight.152403 (PMC8525637; doi:10.1172/jci.insight.152403)
Supplement: Supplemental data [file jciinsight-6-152403-s069.pdf]

SUPPLEMENTAL FIGURES AND TABLES

Human antibody recognition of H7N9 influenza virus hemagglutinin following natural infection

Iuliia M. Gilchuk[1], Sandhya Bangaru[2], Nurgun Kose[1], Robin G. Bombardi[1], Andrew Trivette[1], Sheng Li[3], Hannah L. Turner[4], Robert H. Carnahan[1,5], Andrew B. Ward[4], James E. Crowe, Jr.[1,2,5]

[1]The Vanderbilt Vaccine Center, Vanderbilt Medical Center, Nashville, Tennessee, 37232, USA.
[2]Department of Pathology, Microbiology and Immunology, Vanderbilt University Medical Center, Nashville, Tennessee, 37232, USA.
[3]Department of Medicine, School of Medicine, University of California, San Diego, California, 92093, USA
[4]Department of Integrative Structural and Computational Biology, The Scripps Research Institute, La Jolla, California, 92037, USA.
[5]Department of Pediatrics, Vanderbilt University Medical Center, Nashville, Tennessee, 37232, USA.

SUPPLEMENTAL FIGURES

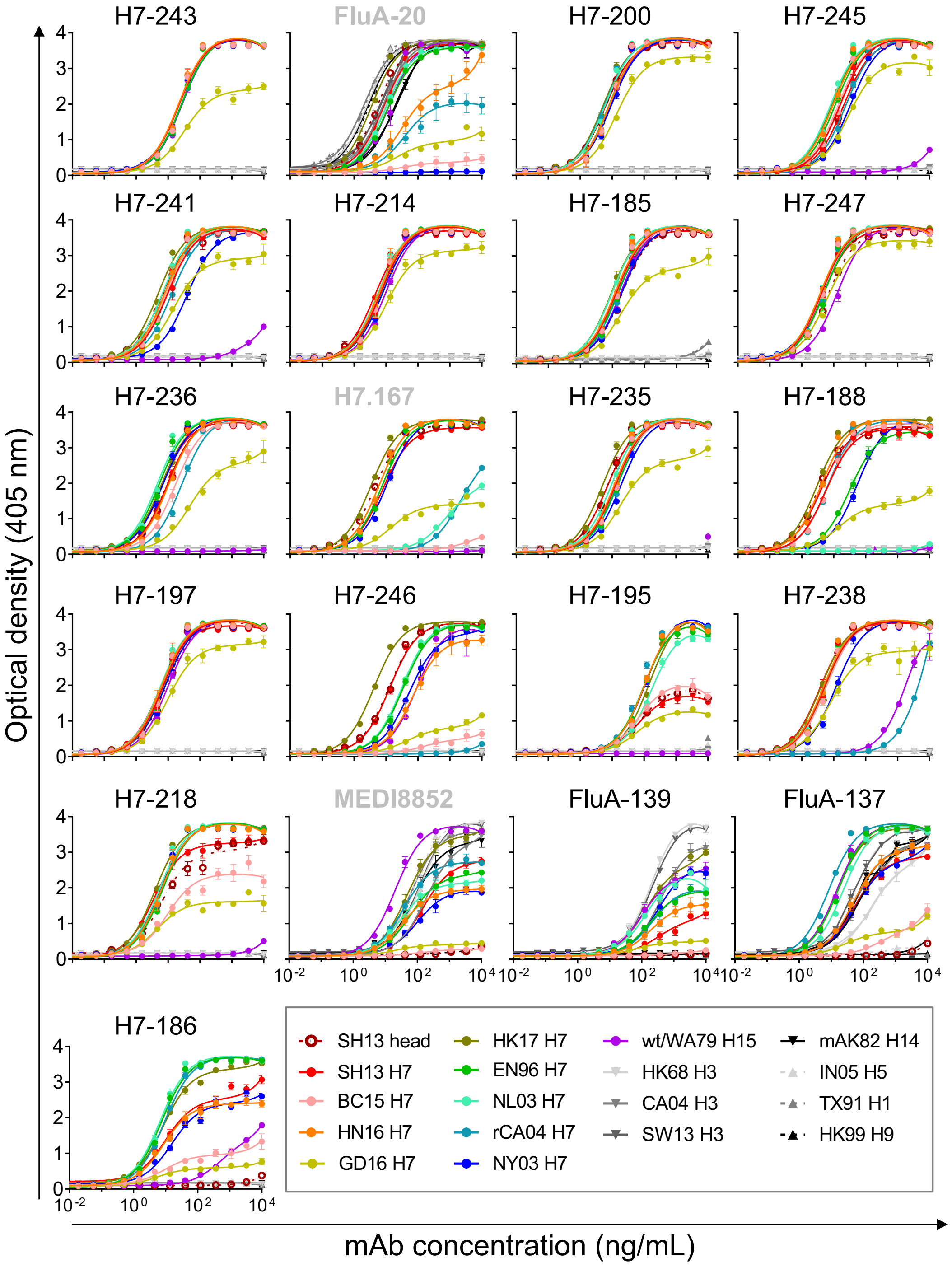

Figure S1. Cross-reactivity of human mAbs to HA antigens. Related to Figure 1. Cross-reactivity of representative mAbs to different HA antigens was assessed by ELISA using purified recombinant HA antigens at a concentration of 1 µg/mL. Gray font color indicates control antibodies. Data represent one of two independent experiments, shown as mean ± SD of assay triplicates.

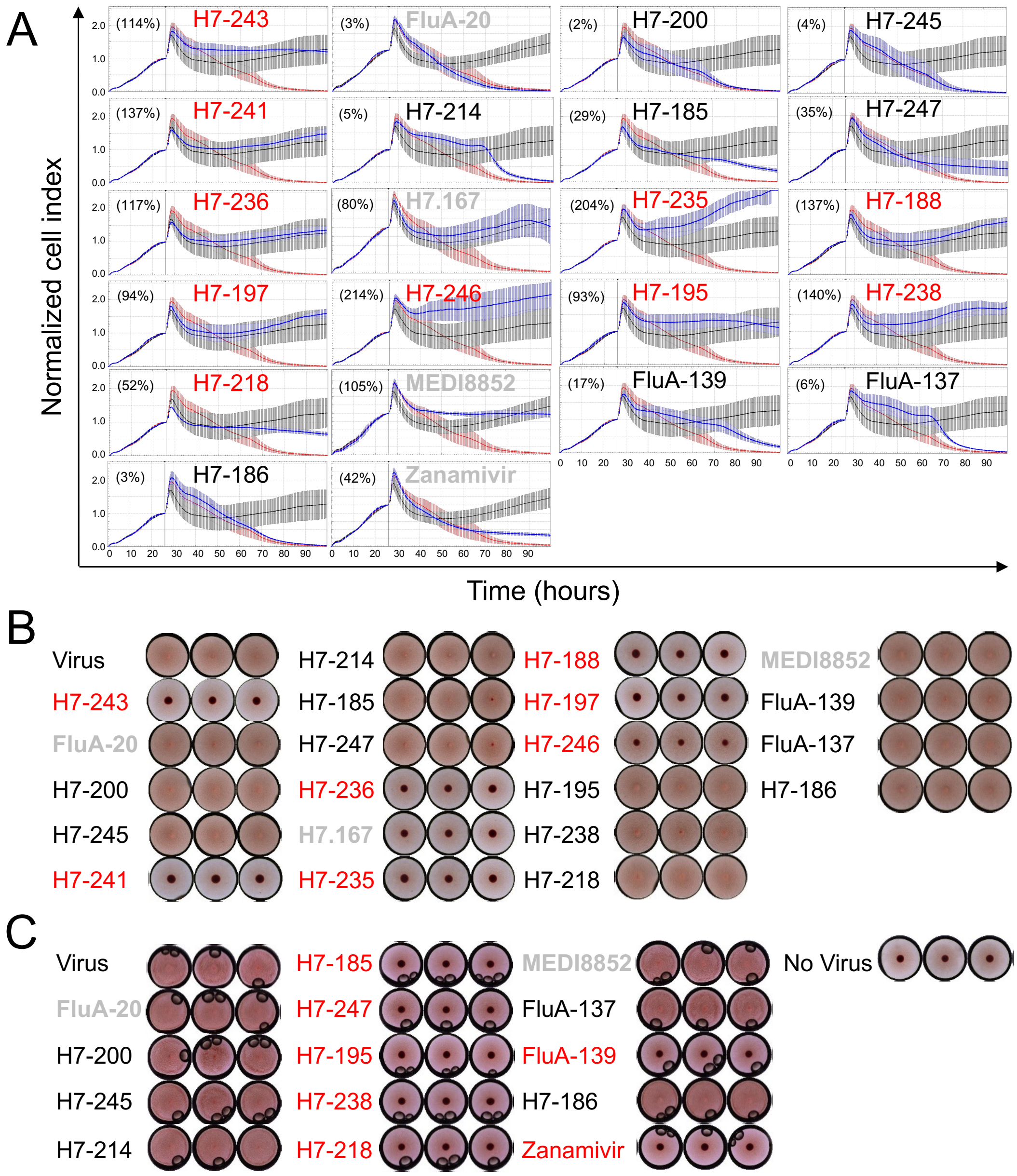

Figure S2. Neutralizing activity characterization of human mAbs from the panel. Related to Figure 2.

Individual mAbs were assessed at a concentration of 10 µg/mL for H7N9 virus neutralization using xCELLigence Real Time Cell Analysis (RTCA) virus-induced cytopathic effect inhibition, hemagglutination inhibition (HAI) and egress inhibition assays.

(A) Neutralizing activity of mAbs measured using RTCA: black curve indicates no viral infection, blue curve indicates virus infection in the presence of mAb, red curve indicates virus infection without mAb. MAb neutralization values are shown as percent in brackets as the mean of three technical replicates.

(B) HAI of virus by mAbs.

(C) Egress inhibition of virus by mAbs.

Red font color indicates neutralizing mAbs. Gray font color indicates control antibodies or inhibitor. Data represent one of two independent experiments with similar results.

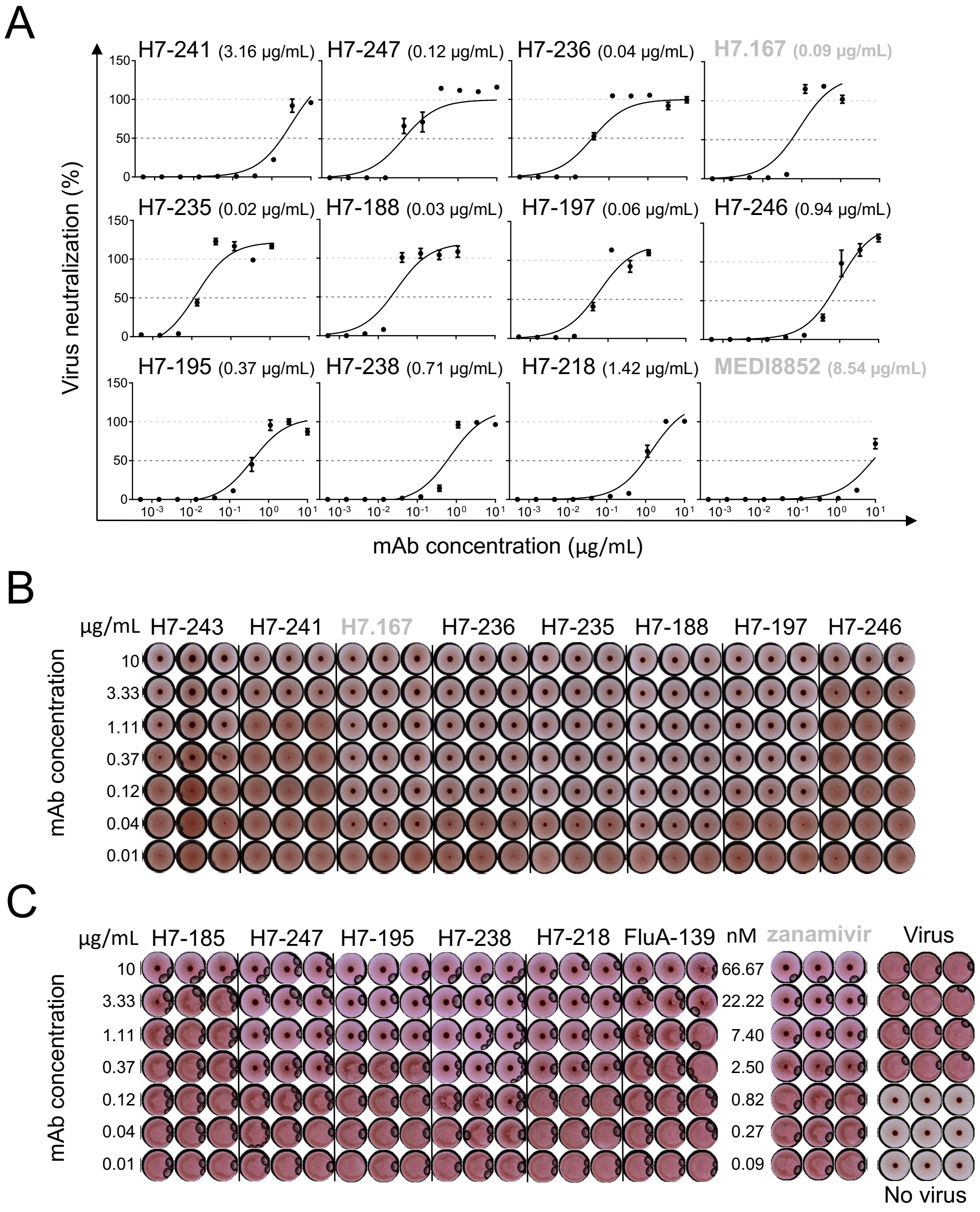

Figure S3. Neutralizing activity of potent mAbs against H7N9 SH13. Related to Figure 2. Neutralizing mAbs were assessed for dose-dependent neutralization of H7N9 virus using xCELLigence Real Time Cell Analysis (RTCA) virus-induced cytopathic effect (CPE) inhibition, hemagglutination inhibition (HAI) and egress inhibition assays.

(A) RTCA virus-induced CPE inhibition assay. IC_{50} values are indicated for each mAb, shown as a mean of assay triplicates.

(B) HAI assay. IC_{100} values are indicated for each mAb, shown as a mean of assay triplicates.

(C) Egress inhibition assay. IC_{100} values are indicated for each mAb, shown as a mean of assay triplicates.

Gray font color indicates control antibodies. Data represent one of two independent experiments with similar results.

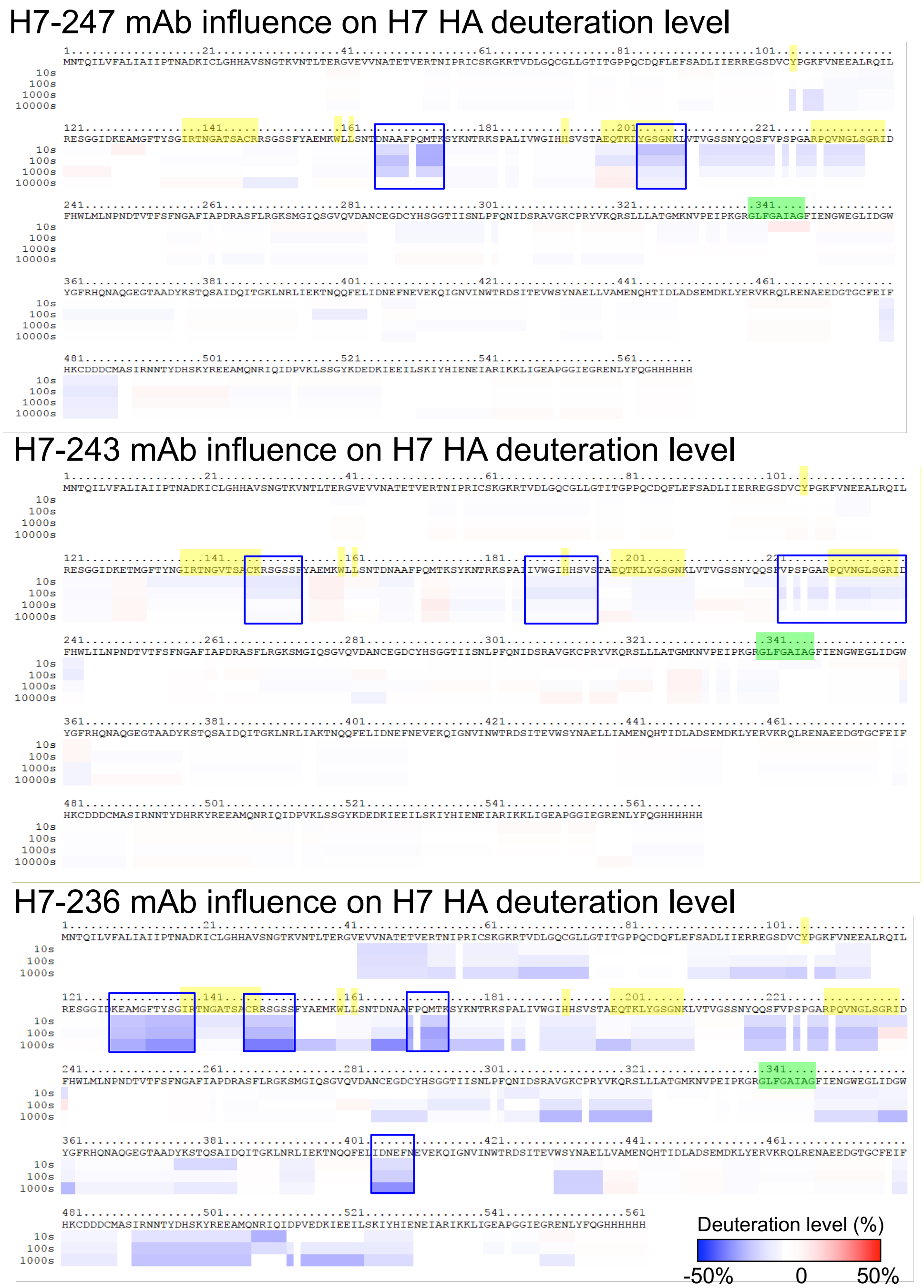

Figure S4. Hydrogen-deuterium exchange mass spectrometry reveals changes in deuteration level for H7 HA incubated with mAbs H7-247, H7-243, or H7-236 compared to H7 HA alone. Related to Figure 3.

Each horizontal row indicates a separate time point (10, 100, or 1,000 seconds [s]). The percentage of deuterium level change is indicated as a color gradient: blue for decrease, red for increase of deuteration level. Blue boxes indicate amino acid residues within the putative epitope, determined by decreased deuteration upon mAb binding to H7 HA. Solid yellow boxes indicate amino acid residues within the H7 receptor binding site. Solid green box indicates the H7 HA fusion peptide.

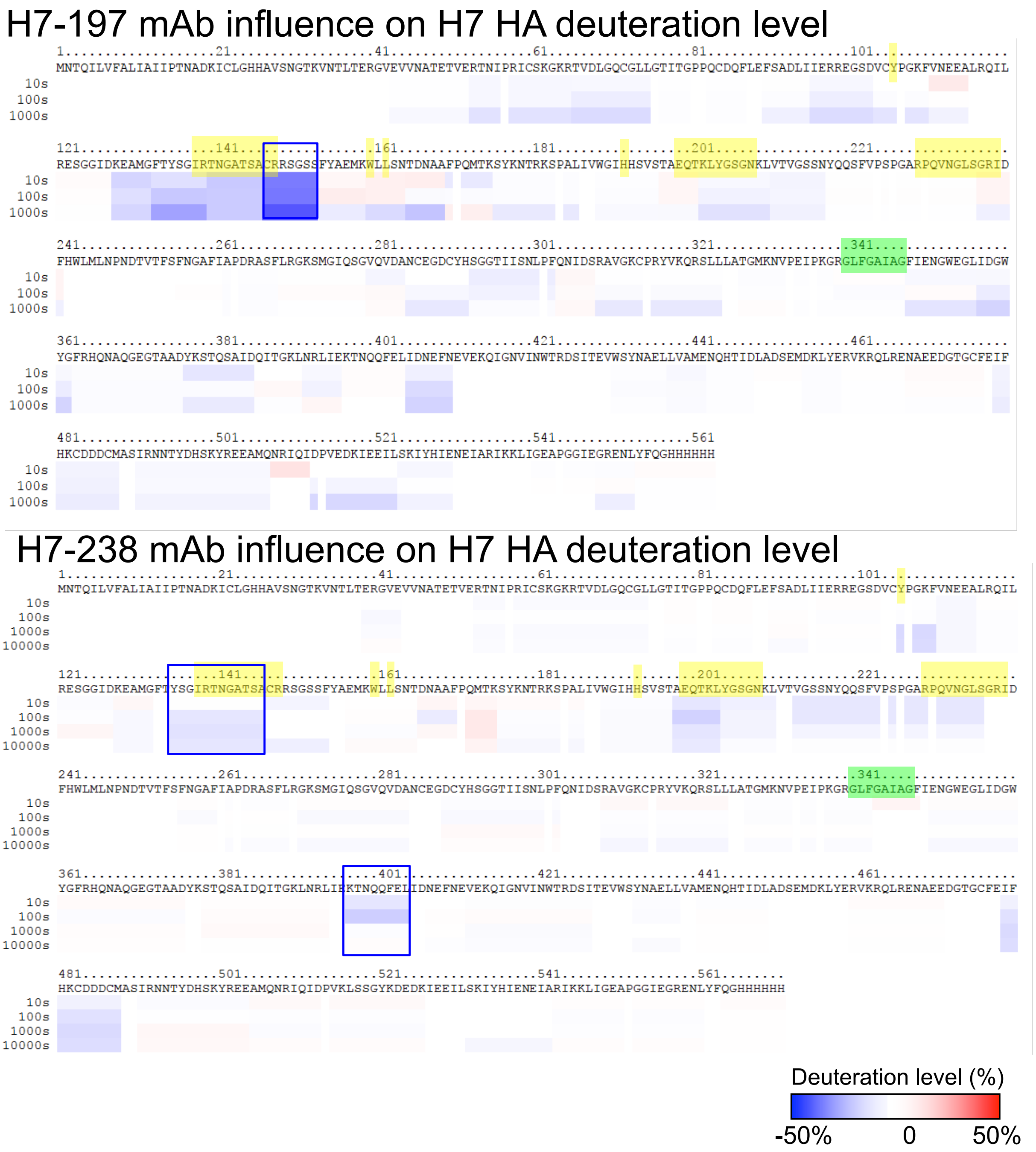

Figure S5. Hydrogen-deuterium exchange mass spectrometry reveals changes in deuteration level for H7 HA incubated with mAbs H7-197 or H7-238 compared to H7 HA alone. Related to Figure 3.

Each horizontal row indicates a separate time point (10, 100, or 1,000 seconds [s]). The percentage of deuterium level change is indicated as a color gradient: blue for decrease, red for increase of deuteration level. Blue boxes indicate amino acid residues within the putative epitope, determined by decreased deuteration upon mAb binding to H7 HA. Solid yellow boxes indicate amino acid residues within the H7 receptor binding site. Solid green box indicates the H7 HA fusion peptide.

SUPPLEMENTAL TABLES

Table S1. Sequence diversity of antibody variable genes encoding H7-specific mAbs, Related to Figure 1.

Donor	mAb	Heavy chain variable gene sequence						Light chain variable gene sequence				
		V_H gene *IGHV-*	V_H region nucleotide % homology to V_H gene	D_H gene *IGHD-*	J_H gene *IGHJ-*	HCDR3 amino acids (aa)	HCDR3 length (aa)	V_L gene *IGLV-*	V_L region nucleotide % homology to V_L gene	J_L gene *IGLJ-*	LCDR3 amino acids (aa)	LCDR3 length (aa)
958	H7-243	*3-30-3*	94	*3-22*	*4*	ARDLWQYLLMGLNY	14	*2-24*	97	*1*	MQATQFPWT	9
957	H7-200	*4-61*	94	*5-18*	*6*	ARESLWNPDYYYYMDV	16	*1-39* or *1D-39*	95	*2*	QQSYSVPYT	9
958	H7-245	*3-33*	97	*1-14*	*6*	TRITGNQHYYGMDV	14	*2-28* or *2D-28*	97	*4*	MQALETLS	8
	H7-241	*3-21*	94	*3-10*	*6*	ARVTAGDSGSYYVPYFYGMDV	21	*2-28* or *2D-28*	98	*4*	MQALQTPALT	10
	H7-214	*3-33*	93	*6-13*	*6*	ARVTAGAGSYHYYGMDV	17	*2-28* or *2D-28*	99	*4*	MEALQSLT	8
957	H7-185	*7-4-1*	95	*5-18*	*4*	ARGGLGFFDF	10	*3-15*	97	*3*	QQYTNWPFT	9
958	H7-247	*4-4*	95	*5-24*	*4*	ASGDGYNFPFEY	12	*3-11*	95	*4*	QQRSRWPPLT	10
	H7-236	*3-21*	95	*2-15*	*4*	ARDLLHHQYCSGGSCFGYFFDY	22	*1-12*	99	*2*	QQANSFPRT	9
	H7-235	*3-33*	96	*6-19*	*6*	ARNGERWRVEDYYYGMDV	18	*2-28*	99	*5*	MQALQTPIT	9
957	H7-188	*ND*	ND	ND	ND	ND	ND	*2-29*	ND	*4*	MLGIHPLT	8
	H7-197	*1-18*	94	*3-16*	*4*	ARDSDYVWGSYFDFRY	16	*1-47*	97	*3*	AAWDDSLSGWV	11
	H7-246	*3-30*	94	*3-9*	*4*	ARAGETYFDWLLPTSDYYMDV	21	*4-1*	98	*4*	QQYYSTPLT	9
	H7-195	*4-39*	95	*6-19*	*4*	ATSGRIAVAGTGIEN	15	*3-1*	96	*1*	QAWDTSTVYV	10
958	H7-238	*1-18*	97	*6-19*	*5*	ARSTGGGSSSWFDP	14	*3-15*	100	*3*	QQYNNWLFT	9
	H7-218	*3-21*	99	*2-15*	*3*	TRDLGRGLELGPSAFDI	17	*1-36*	99	*1*	AAWDDSLNGYV	11
	FluA-139	*1-46*	94	*3-9*	*5*	ARTPASNNILTGVLALNYFGP	21	*4-1*	99	*3*	QQYYSNPIT	9
	FluA-137	*3-30-3*	97	*6-13*	*6*	ARDKTIAAAGYYYGLDV	17	*1-39*	98	*2*	QQTYSTLMCT	10
957	H7-186	*3-66*	96	*1-7*	*3*	ARGFNWNYVGAFDV	14	*3-21*	97	*3*	QVWDSSSDHWV	11

ND indicates not determined.

Table S2. Cross-reactivity of H7-reactive mAbs, Related to Figure 1

Donor	Isotype	mAb	EC50 (ng/mL) for binding																										
			HA antigens from Group 2																HA antigens from Group 1										
			H7										H15	H3			H14	H4	H6	H11	H2	H5		H1			H8	H12	H9
			$SH13_{head}$*	SH13	BC15	HN16	GD16	HK17	EN96	NL03	rCA04	NY03	wtsWA79	HK68	CA04	SW13	mAK82	dCS56	TW13	dMP74	SG57	VN04	IN05	PR34	TX91	CA09	tOT68	dAB76	HK99
958	IgG3	H7-243	ND	20	21	19	33	ND	24	20	24	21	25	>	>	>	>	>	>	>	>	>	ND	>	>	ND	>	>	>
N/A	IgG1	FluA-20	5	8	>	56	50	3	11	9	36	>	11	10	8	6	18	2	3	100	15	371	2	1	2	5	2	2	3
957	IgG1	H7-200	6	6	8	8	13	5	7	6	5	9	8	>	>	>	>	>	>	>	>	>	>	>	>	>	>	>	>
958	IgG1	H7-245	14	19	13	9	27	8	10	7	22	31	>	>	>	>	>	>	>	>	>	>	>	>	>	>	>	>	>
	IgG1	H7-241	7	6	8	7	15	5	9	6	12	39	>	>	>	>	>	>	>	>	>	>	>	>	>	>	>	>	>
	IgG1	H7-214	6	4	6	6	10	6	7	5	6	7	9	>	>	>	>	>	>	>	>	>	>	>	>	>	>	>	>
957	IgG1	H7-185	18	12	12	11	20	8	13	9	14	18	17	>	>	>	>	>	>	>	>	>	>	>	>	>	>	>	>
958	IgG1	H7-247	6	4	4	4	6	4	5	4	5	4	12	>	>	>	>	>	>	>	>	>	>	>	>	>	>	>	>
	IgG3	H7-236	9	6	15	10	58	6	5	4	25	6	>	>	>	>	>	>	>	>	>	>	>	>	>	>	>	>	>
N/A	IgG1	H7.167	4	8	>	6	15	3	7	>	>	10	>	>	>	>	>	>	>	>	>	>	>	>	>	>	>	>	>
958	IgG1	H7-235	7	7	12	11	20	5	13	9	14	18	>	>	>	>	>	>	>	>	>	>	>	>	>	>	>	>	>
957	IgG1	H7-188	3	4	6	4	21	3	30	>	6	51	>	>	>	>	>	>	>	>	>	>	>	>	>	>	>	>	>
	IgG1	H7-197	8	5	6	6	10	6	7	5	6	7	9	>	>	>	>	>	>	>	>	>	>	>	>	>	>	>	>
	IgG1	H7-246	12	13	307	69	101	4	32	29	>	54	82	>	>	>	>	>	>	>	>	>	>	>	>	>	>	>	>
	IgG1	H7-195	63	57	52	91	55	126	85	150	67	101	>	>	>	>	>	>	>	>	>	>	>	>	>	>	>	>	>
958	IgG1	H7-238	5	4	5	4	8	4	5	4	9460	11	1562	>	>	>	>	>	>	>	>	>	>	>	>	>	>	>	>
	IgG1	H7-218	7	4	9	6	5	4	6	5	6	6	>	>	>	>	>	>	>	>	>	>	>	>	>	>	>	>	>
N/A	IgG1	MEDI8852	>	152	>	36	>	49	64	31	37	96	19	119	208	112	89	69	ND	>	>	64	70	24	62	30	163	>	>
958	IgG1	FluA-139	>	>	>	197	>	176	175	104	201	290	119	175	401	202	>	>	>	>	>	>	>	>	>	>	>	>	>
	IgG1	FluA-137	>	58	>	52	58	15	16	22	8	74	14	233	90	55	70	>	>	>	>	>	>	>	>	>	>	>	>
957	IgG3	H7-186	>	15	19	9	8	9	7	6	9	18	>	>	>	>	>	>	>	>	>	>	>	>	>	>	>	>	>

* indicates A/Shanghai/02/2013 HA head domain monomer;
ND indicates not determined;
The > symbol indicates binding was not detected when tested at concentrations as high as 10 µg/mL;
N/A indicates not applicable.
